# Microbial Contamination of Seven Major Weaning Foods in Nigeria

**DOI:** 10.3329/jhpn.v29i4.8459

**Published:** 2011-08

**Authors:** F. Oluwafemi, I. Nnanna Ibeh

**Affiliations:** ^1^Department of Microbiology, University of Agriculture, Abeokuta, Nigeria; ^2^Department of Microbiology, Faculty of Life Science, University of Benin, Benin city, PMB 1154, Nigeria

**Keywords:** Aflatoxins, High-performance liquid chromatography, Microbial contamination, Microbial count, Weaning foods, Nigeria

## Abstract

Five million children aged less than five years die annually due to diarrhoea. The aim of the study was to identify some possible contributing factors for persistent diarrhoea. Seven weaning foods, including a locally-made food, were evaluated by estimating the microbial load using the most probable number method and aflatoxin levels (AFM_1_, AFG_1_, AFG_2_, and AFB_2_) by immunoaffinity column extraction and high-performance liquid chromatography (HPLC) with detection of fluorescence. The results showed that the locally-made weaning food had the highest microbial count (2,000 cfu/g) and faecal streptococcal count (25 cfu/g). Moulds isolated were mainly *Aspergillus niger, A. flavus, A. glaucus, Cladosporium* sp., and *Penicillium* sp. The home-made weaning food recorded the highest fungal count (6,500 cfu/g). AFM_1_ of the weaning foods was 4.6-530 ng/mL. One weaning food had AFB_1_ level of 4,806 ng/g. Aflatoxin metabolites, apart from AFM_1_ and AFB_1_ present in the weaning foods, were AFG_1_ and AFG_2_. There were low microbial counts in commercial weaning foods but had high levels of aflatoxins (AFM_1_, AFG_1_, AFG_2_, AFB_1_, and AFB_2_). Growth and development of the infant is rapid, and it is, thus, possible that exposure to aflatoxins in weaning foods might have significant health effects.

## INTRODUCTION

Microbial contamination leading to infections and poor nutrient associated with weaning foods may contribute significantly to deaths of 13 million infants and children aged less than five years worldwide each year (1,2). After respiratory infections, diarrhoeal diseases are the commonest illness and have the greatest negative impact on the growth of infants and young children (3,4). The causes of diarrhoeal diseases have traditionally been ascribed to water supply and sanitation (5). To prevent such diseases, governments and non-governmental organizations have focused their efforts on and sometimes limited to improving water supply and sanitation and promoting and protecting breastfeeding with less emphasis on food safety. This issue is increasingly becoming important in national and international debates about agriculture, nutrition, and health. Food safety is not a luxury of the rich but a right of all people (6). Based on literature, weaning foods prepared under unhygienic conditions are frequently heavily contaminated with pathogens and may, thus, be a major factor in causing diarrhoeal diseases and associated malnutrition. In particular, traditional gruels used in The Gambia for supplementing breastmilk were found to be heavily contaminated with potentially pathogenic micro-organisms, and such supplements are important factors in weaning-related diarrhoea (7). Therefore, it appears that current efforts are not sufficient to prevent diarrhoeal diseases; thus, education of mothers in food-safety principles, particularly weaning foods, must also receive high priority (2). Educational programmes based on the hazard-analysis-critical-control-point approach, taking into consideration also sociocultural factors, should be integrated into all national infant-feeding or food and nutrition programmes (8).

Food-borne infections can have dangerous and long-term effects, especially on nutritional status. Formula-fed infants usually require formula for their first year but they are introduced to other kinds of foods once they reach six months of age. Most food items used for the composition of weaning foods, such as groundnuts, maize, and other oilseeds, are vulnerable crops to moulds, especially *Aspergillus parasiticus* and *A. flavu*s that produce aflatoxins (AFs) (9-11). These toxigenic fungi grow well. However, it is more serious in tropical countries of the world where humidity is high and the temperature is conducive for the growth and production of AFs. AFs are potent carcinogens, mutagens, teratogens, and immunosuppressants. In addition to being potent carcinogens, AFs may contribute to early growth faltering of the child (12), and strong associations have been reported around the weaning stage in Beninese infants (13,14). A study in Beninese children reported that secretory IgA in saliva may be reduced by dietary levels of AF (13). The immune status of Ghanaian adults has been reportedly affected by exposure to AFs (15). The aim of this study was to ascertain the microbiological and AF status of some weaning foods by screening such foods sold in Nigeria.

## MATERIALS AND METHODS

### Sample collection

Seven weaning foods collected from open markets in Ibadan, Nigeria, and one locally-made weaning food voluntarily donated were examined for the microbial load and AF levels. The major ingredients of the weaning foods were as follows: weaning food A was made of milk and wheat; weaning food B was made of rice and milk; weaning food C was made of maize and soya; weaning food D, E, and F were made of maize and milk; and weaning food G was made of maize, fish, groundnut, and soya.

### Microbiological analysis

The total microbial load was determined using nutrient agar prepared according to the guidelines of the manufacturer. Serial dilution was done using physiological salt solution containing NaCl and NaHPO4 (1.45 g, 10 g, and 6.25 per 2.5 L) as diluents. The aim was to maintain the microorganisms in their physiological state to prevent plasmolysis resulting from osmosis. One hundred mL of the diluent was measured into bottles used for serial dilution containing 11 g of each sample. The mixture was shaken using a horizontal shaker (Model SM, Einrichtungen, Germany) for 30 minutes. Further dilutions were made, and dilution 10^1^ and 10^3^ were plated in duplicates.

Faecal coliform counts were determined using 36.5 g/L of fluorocult media containing durham tubes. The most probable number (MPN) was used, and 10^1^, 10^2^, and 10^3^ dilutions were plated out. Any tube containing gas was an indication of the presence of faecal coliform. The next stage was to confirm faecal coliform by adding 1/2 mL of NaOH to tubes containing gas. Sodium hydroxide will neutralize the acid and enable the media to fluoresce if faecal coliforms are present.

Fungal counts were determined using Dichloran Glycerin (DG) 18. One mL of streptomycin (650 mg/100 mL membrane filtered) was added to the media, and dilution 10^1^ and 10^3^ were plated in duplicates.

The nutrient agar bacteria plates were incubated at 37 ^o^C for 48 hours, and the fungi plates were incubated at room temperature (28 ^o^C) for five days.

### Aflatoxin analysis of weaning foods

Chemicals and solvents used were of HPLC grade or equivalent. All water used was distilled and, for HPLC, passed through a Milli-Q purification system (Millipore, London, UK). Acetonitrile used for mobile phase was of HPLC grade and provided by Merck, Darmstadt, Germany.

### Analysis

Immunoaffinity columns (IACs) (RIDA aflatoxin column, R-BIOPHARM, Darmstadt) were used for cleaning the sample extracts. The IAC was brought to room temperature, plugged unto Luer-attachment of a vacuum pressure facility. Twenty-five g of each weaning food was weighed into a 250-mL Erlenmeyer flask, 125 mL of acetone/water (85:15, v/v) was added and placed on a magnetic stirrer (Telemodule, Labortechnik) at 500 rpm for 45 minutes, and 5 mL of the filtrate was measured into a vessel connected to the IAC. Forty-five mL of Millipore water was added to the barrel attached to the IAC. The sample was allowed to flow through the IAC at a rate of ca. 1 mL per minute. Slight pressure was applied. The reservoir was rinsed twice with 10 mL of phosphate buffer at a flow rate of ca. 2-3 mL per minute. Then the reservoir was removed, and the IAC was dried by applying pressure. AFM_1_ was collected in glass bottles previously treated with 2N H_2_SO_4_. The solvent used for eluting was 1.25 of mL methanol/acetonitrile (20:30, v/v). The eluate was dried in a water bath at 40 ^o^C and 80 kPa. The dried extract was re-dissolved with 1.25 mL of acetonitrile:water (25:75, v/v). The acetonitrile/water solution was used as the mobile phase for HPLC. AFB_1_, AFB_2_, AFG_1_, and AFG_2_ were also determined.

### **Determination of aflatoxins by HPLC**

The HPLC system consisted of a LDC, Milton Roy, Consta Metric 1 pump, and a Lichrosorb RP-18 (Merck Hibar) column (particle size of 5 µm, length–125 mm, inside diameter–4 mm). The pump pressure was 60 MPa. The injector was an automatic type (Rheotype Gilson Abimed Model 231). The detector had a fluorescence spectrophometer (Shimadzu RF 535, gamma excitation–365 mm and gamma emission–444 nm). The flow rate was 1 mL per minute, and the injection volume was 50 µL. The mobile phase was water/acetonitrile (75:25).

### Standard solutions

AFM_1_, AFB_1_, AFB_2_, AFG_1_, and AFG2 were obtained from Sigma-Aldrich (St. Louis, MO, USA). The commercial stock solution of AFM_1_ was 1,000 ng/mL. The spike solution was made by diluting the stock solution 1:40 to give approximately 25 ng/mL using HPLC grade acetonitrile/water. Of the diluted stock solution, 140 µL was added to 70 mL of defatted Hipp baby milk. Calibration curve was prepared by diluting 2 µg/L of AFM_1_ in a 1:500 dilution.

The stock solutions were stored at 4 ^o^C when not in use.

### Validation and repeatability of measurements

Validation and repeatability of measurements were done using the Hipp Baby formula bought from a supermarket in Munich, Germany. Seventy g of powdered milk was weighed into a one-litre beaker, and 450 mL of Millipore water added. The mixture was stirred vigorously with a stirrer. The temperature of milk was raised to 50 ^o^C to ensure proper dispersion of milk fat. Thereafter, the milk temperature was lowered to room temperature. Four centrifuge tubes were half-filled and centrifuged at 3,000 g for 15 minutes. To aid the removal of fat, the milk samples were kept in the cold room (4 ^o^C) for 15 minutes. The solidified fat was scooped off, and the milk samples were filtered using ashless filter circles (MN 640W, diameter–150 mm) (Macherey-Nagel, Germany). Seventy mL of defatted Hipp milk was measured into five 100-mL volumetric flasks. Three of the flasks containing Hipp-defatted milk were spiked with 25 ng/mL AFM_1_, and two samples served as controls. AFM_1_ was extracted as previously described above. The average recovery rates were calculated. AFB_1_, AFB_2_, AFG_1,_ and AFG_2_ were also determined using standard AFB_1_, AFB_2_, AFG_1_, and AFG_2_ as reference.

**Table 1.** Validation and repeatability of measurement using Hipp baby formula by HPLC

Sample name	Ret time min	Area Mv min	Height Mv	Amount ppb	% of recovery
Std 2.0 ppb	5.48	221,323	878,118	2.00001	100
Std 1.0 ppb	5.49	110,008	43,988	1.0003	100
Std 0.5 ppb	5.47	54,896	219,905	0.4985	99.7
Std 0.25 ppb	5.48	27,419	111,156	0.2504	100.16
Spiked milk					
2.5 ppb (1)	5.57	55,298	246,355	2.23	89
(2)	5.57	56,604	256,889	2.33	93
(3)	5.57	59,533	27,180	2.58	103

HPLC=High-performance liquid chromatography;

min=Minutes;

Mv=Measurement unit;

Ret=Retention;

Std=Standard; 1, 2, and 3 with 2.5 ppb indicate replicate 1, 2, and 3

**Table 2.** Aflatoxin levels (ng/mL) of some weaning foods sold in Nigeria

Sample	AFM_1_	AFB_1_	AFB_2_	AFG_1_	AFG_2_
WFA	127.6	-	464.0	-	1,699
WFB	4.6	-	8,290	-	1,169
WFC	-	4,806	-	-	-
WFD	-	-	-	-	-
WFE	530	-	387	144	-
WFF	-	181.6	103	-	-
WFG	-	-	-	-	-

Indicates not detected;

Af=Aflatoxin;

WF=Weaning food

**Table 3.** Microbiological analysis of some weaning foods sold in Nigeria

Weaning food/major ingredient	Total viable count (cfu/g)	Faecal coliform count (cfu/g)	Fungal count (cfu/g) and species
WFA (wheat+milk)	2.2×10^6^	<2	10, *Cladosporium* sp.
WFB (rice+milk)	No growth after 72 hours	<2	10, *Cladosporium* sp.
WFC (maize+soya)	No growth after 72 hours	<2	15, *Penicillium* sp.
WFD (maize+milk)	20	<2	30, *A. flavus* and *Cladosporium* sp.
WFE (maize+milk)	50	<2	50, *A. flavus Cladosporium* sp.
WFF (maize+milk)	500	<2	15, *A. niger*, Mucor
WFG (maize, groundnut, fish +soya)	2×10^3^	25	6,500, *A glacus*, *A*. *niger*, and *A. flavus*

WF=Weaning food

### Statistical analysis

Comparison of statistical analysis between means was evaluated by Student's *t*-test and analysis of variance. A value of p<0.05 was considered significant.

## RESULTS

Seven weaning foods were bought from an open market, including a locally-home made weaning food, which was voluntarily donated. These foods were examined for AFM_1_, AFB_1_, AFB_2_, AFG_1_, and AFG_2_ by immunoaffinity column extraction and HPLC with detection of fluorescence. AFM_1_ was detected in three samples. Two samples were above the 500 ng/g AFM_1_ approved by the Nigeria National Agency for Food and Drug Administration and Control while AFB_1_, AFB_2_, AFG_1_, and AFG_2_ were found in five samples. Three weaning foods had AFB_1_ ranging from 181.6 to 4,806 ng/g (Table 2). One sample had AFB_1_ level of 4,806 ng/g, and another baby food had AFM_1_ of 530 ng/g. Only one sample and the locally-made weaning food had no AFs. AF metabolites present in weaning foods, apart from AFB_1_ and AFM_1_, were AFG_1_ and AFG_2_. All positive samples had extremely high AFB_1_, AFB_2_, AFG_1_, and AFG2. The main raw ingredients of the weaning foods were maize, cassava, yam, melon, groundnuts, and foods rich in mycotoxins.

Microbiology of the weaning foods revealed low microbial counts in commercial weaning foods but high AF levels (AFM_1_, AFG_1_, AFG_2_, AFB_1_, and AFB_2_). The results showed that the home-made weaning food had the highest microbial count (2,000 cfu/g) and faecal streptococcal count (25 cfu/g) using MPN in a fluorocult medium (Table 3). Moulds isolated were mainly *A. niger, A. flavus, A. glaucus, Cladosporium* sp., and *Penicillium* sp., and the home-made weaning food recorded the highest fungal count of 6,500 cfu/g.

## DISCUSSION

Children are a highly-susceptible population group for exposure to environmental toxicants for various reasons, including lower detoxification capacity, rapid growth, higher intakes of air, food, and water per kg of body-weight (16), and early childhood exposure to bacterial and carcinogenic AFs may, therefore, be the critical determinants of immediate and later health effects.

The bacterial and fungal counts of most commercial weaning foods sold in Nigeria were low probably due to good food-handling and good manufacturing practices. Fungal counts may be low in processed foods but not AF levels since AFs cannot be destroyed by normal cooking temperature (17). Table 2 shows that bacterial and fungal counts were low but heat-processed commercial weaning foods had unacceptable high levels of AFB_1_, AFB_2_, AFG_1_, and AFG2. Exposure in early infancy is occurring at levels that are not safe for the development of the child.

In developing countries, such as Nigeria, growth faltering is often associated with the quantity and/or poor quality of foods, in addition to multiple infectious hazards (18). However, high levels of AF-albumin adducts have been associated with growth faltering in Beninese infants (13,14). Egyptian infants had a high prevalence of stunting and moderate frequency of being underweight, based on the criteria of the World Health Organization (19,20). The exposure of children to AFs may be high in Nigeria. Genotoxic, carcinogenic, immunosuppressive, teratogenic substances, such as AFs, do not have a threshold value for human health below which the risk value is equal to zero. The Joint FAO/WHO Expert Committee on Food Additives does not have tolerable daily intake (TDI) of AF. This simply means that no level of AF is safe from the toxicological point of view but strongly recommends that the AF level should be as low as possible (16). Therefore, the toxicological significance of the presence of AFs in foods should not be overlooked. To reduce the exposure of infants to AFs, education of mothers is highly recommended. A reduction in AF levels in weaning foods is desirable. Reductions in exposure to AFs can be achieved by several approaches. In Nigeria, the source of contamination is clearly defined, such as poor post-harvest handling and storage of risk foods (13). In addition to controlling post-harvest changes, dietary modulation, e.g. with chlorophyllin (21) or probiotics (22,23), antioxidants, such as selenium and vitamins (24), are effective.

## References

[1] World Health Organization (1993). Food Safety Unit. Contaminated food: a major cause of diarrhoea and associated malnutrition among infants and young children.

[2] Motarjemi Y, Kaferstein F, Moy G, Quevedo F (1993). Contaminated weaning food: a major risk factor for diarrhoea and associated malnutrition. Bull World Health Organ.

[3] Lee MB, Middleton D (2003). Enteric illness in Ontario, Canada, from 1997 to 2001. J Food Prot.

[4] Tetteh IK, Frempong E, Awuah E (2004). An analysis of the environmental health impact of the Barekesse Dam in Kumasi, Ghana. J Environ Manage.

[5] Rowland MG, Barrel RA, Whitehead RG (1978). Bacterial contamination in traditional Gambian weaning foods. Lancet.

[6] World Health Organization UN Food and Trade Standards Commission opens meeting to adopt new standards for foods and revise others, 2003.

[7] Iroegbu CU, Ene-Obong HN, Uwaegbuta AC, Amazigo UV (2000). Bacteriological quality of weaning food and drinking water given to children of market women in Nigeria: implications for control of diarrhoea. J Health Popul Nutr.

[8] Blais BW, Leggate J, Bosley J, Martinez-Perez A (2005). Detection of Escherichia coli 0157 in foods by a novel polymyxin-based enzyme-linked immunosorbent assay. J Food Prot.

[9] Faletto MB, Koser PL, Batula N, Townsend GK, Maccubbin AE, Gelboin HV (1988). Cytochrome P3–450 cDNA encodes aflatoxin B_1_ hydrolase. J Biol Chem.

[10] Kim EK, Shon DH, Ryu D, Park JW, Hwang HJ, Kim YB (2000). Occurrence of aflatoxin M_1_ in Korean dairy products determined by ELISA and HPLC. Food Addit Cont.

[11] Jimoh KO, Kolapo AL (2008). Mycoflora and aflatoxin production in market samples of some selected Nigerian foodstuffs. Res J Microbiol.

[12] Turner PC, Moore SE, Hall AJ, Prentice AM, Wild CP (2003). Modification of immune function through exposure to dietary aflatoxin in Gambian children. Environ Health Perspect.

[13] Gong Y, Cardwell K, Hounsa A, Egal S, Turner PC, Hall AJ (2002). Dietary aflatoxin exposure and impaired growth in young children from Benin and Togo: cross sectional study. BMJ.

[14] Gong Y, Hounsa A, Egal S, Turner PC, Sutcliffe AC, Hall AJ (2004). Postweaning exposure to aflatoxin results in impaired child growth: a longitudinal study in Benin, West Africa. Environ Health Perspect.

[15] Jiang Y, Jolly PE, Ellis WO, Wang JS, Phillips TD, Williams JH (2005). Aflatoxin B1 albumin adduct levels and cellular immune status in Ghanaians. Int Immunol.

[16] Safety evaluation of certain mycotoxins in food (2001). Prepared by the fifty-sixth meeting of the Joint FAO/WHO Expert Committee on Food Additives.

[17] Carvajal M, Bolanos A, Rojo F, Mendez I (2003). AflatoxinM1 in pasteurized and ultra-pasteurized milk with different fat content in Mexico. J Food Prot.

[18] World Health Organization (2003). Global strategy for infant young child feeding.

[19] Polychronaki N, Turner PC, Mykkanen H, Gong Y, Amra H, Abdel-Wahab M (2006). Determinants of aflatoxin M1 in breast milk in a selected group of Egyptian mothers. Food Addit Contam.

[20] World Health Organization (2003). Global strategy for infant and young child feeding.

[21] Egner PA, Wang JB, Zhu YR, Zhang BC, Wu Y, Zhang QN (2001). Chlorophyllin intervention reduces aflatoxin-DNA adducts in individuals at high risk for liver cancer. Proc Natl Acad Sci USA.

[22] El-Nezami HS, Polychronaki NN, Ma J, Zhu H, Ling W, Salminen EK (2006). Probiotic supplementation reduced biomarker for increased risk of liver cancer in young Chinese males from southern China. Am J Clinical Nut.

[23] Oluwafemi F, Ikeowa MC (2005). Fate of aflatoxin B_1_ during fermentation of maize into “Ogi”. Nig Food J.

[24] Chen H, Tappel AIL (1996). Protection of multiple antioxidants against heme protein oxidation and lipid peroxidation induced by CBrCl_3_ in liver, lung, heart and spleen. J Agric Food Chem.

